# Long-term clinical and radiographic outcomes of anatomic total shoulder arthroplasty with a short-stem humeral component: a ten-year follow-up study

**DOI:** 10.1007/s00264-026-06920-1

**Published:** 2026-07-17

**Authors:** Fabian Lanzerath, Markus Loew, Julia Bockmeyer, Dominik Ferrari, Marc Schnetzke

**Affiliations:** 1 Department of Orthopedic and Trauma Surgery, University Hospital Cologne, Cologne, Germany; 2 Present Address: Department of Orthopaedics, Heidelberg University Hospital, Heidelberg, Germany; 3 Department of Shoulder and Elbow Surgery, Atos (Germany), Heidelberg, Germany; 4 Department of Orthopedic and Trauma Surgery, Berufsgenossenschaftliche Unfallklinik Ludwigshafen, Ludwigshafen am Rhein, Germany; 5 Department of Trauma and Reconstructive Surgery, St. Vincentius-Kliniken, Karlsruhe, Germany

**Keywords:** Anatomic shoulder arthroplasty, Short-stem, Long-term follow-up, Humeral bone remodeling, Stress shielding, Glenoid loosening

## Abstract

**Purpose:**

This study aimed to provide long-term clinical and radiographic follow-up data for anatomic total shoulder arthroplasty using a short-stem humeral component with a cemented keeled glenoid in primary glenohumeral osteoarthritis.

**Methods:**

Between 2010 and 2013, 35 consecutive anatomic total shoulder arthroplasties were performed at a single institution by one surgeon. At a mean follow-up of 10.3 ± 1.1 years, seven patients had died and five were lost, leaving 23 shoulders (82% of surviving cases) available for independent clinical and radiographic analysis. Clinical evaluation included the Constant Score, age- and sex-adjusted Constant Score, Subjective Shoulder Value, pain, and range of motion. Standardized radiographs at four time points were assessed for humeral remodeling, glenoid radiolucency, and secondary rotator cuff insufficiency.

**Results:**

Clinical outcomes improved significantly compared with baseline and remained stable through long-term follow-up. The mean Constant Score improved from 29.5 ± 5.6 preoperatively to 72.4 ± 7.4 at final follow-up, with 16 shoulders (69.6%) graded as excellent. Radiographically, moderate-to-severe humeral remodeling was present in 11 shoulders (47.8%) at final follow-up, but remodeling was not associated with inferior clinical outcomes. Secondary rotator cuff insufficiency, assessed indirectly by acromiohumeral distance, was present in seven shoulders (30.4%). Higher glenoid radiolucency was associated with reduced forward elevation and abduction. One patient (4.3%) underwent revision 13.2 years after the index procedure due to aseptic glenoid loosening with secondary cuff insufficiency and was converted to reverse total shoulder arthroplasty.

**Conclusion:**

Anatomic total shoulder arthroplasty with a cemented keeled glenoid and a bone-preserving short-stem humeral component provided durable pain relief and functional improvement beyond ten years in this cohort. Humeral remodeling was frequent but clinically well tolerated, whereas glenoid radiolucency appeared to be associated with inferior long-term functional parameters and represented the only cause for revision in this cohort.

## Introduction

Anatomic total shoulder arthroplasty (aTSA) has demonstrated reliable long-term outcomes for the treatment of primary glenohumeral osteoarthritis [1, 2]. Traditional cemented long-stem implants offer durable fixation but pose significant challenges during revision, including extensive bone loss [3, 4]. In response, uncemented short-stem and stemless designs have gained popularity, aiming to preserve bone stock, facilitate revision, and more closely replicate native load transfer to the proximal humerus [5–8].

Biomechanical analyses have supported these theoretical advantages, showing that shorter stems reduce stress shielding and better maintain physiologic bone stress distribution in the proximal humerus compared with standard-length stems [5]. However, short-stem implants have also been associated with specific radiographic changes, including cortical thinning and medial calcar osteolysis, which raise concerns about long-term implant stability [9–11].

Short-term and mid-term studies have reported encouraging clinical outcomes after short-stem aTSA, whereas truly long-term data beyond ten years remain scarce and are often difficult to compare because cohorts include different stem lengths, implant generations, glenoid designs, and follow-up intervals [7]. In addition, the clinical relevance of humeral remodeling after short-stem fixation remains insufficiently defined. Since late failure after aTSA may also be related to the glenoid component or progressive rotator cuff dysfunction, glenoid radiolucency and secondary rotator cuff insufficiency were included as major secondary radiographic endpoints in the present study.

We asked whether aTSA for primary glenohumeral osteoarthritis using an uncemented short-stem humeral component (Ascend Monolithic) and a cemented keeled glenoid provides durable clinical improvement at a mean ten-year follow-up (primary outcome: Constant Score) and whether long-term radiographic findings, including humeral remodeling, glenoid radiolucency and secondary rotator cuff insufficiency, are associated with clinical outcome. We hypothesized sustained improvement, with glenoid radiolucency as the principal adverse radiographic correlate.

## Materials and methods

### Study design

This was a retrospective single-centre consecutive case series. Between October 2010 and February 2013, a consecutive series of 35 shoulder arthroplasties in 34 patients was performed by one fellowship-trained shoulder surgeon at a single specialised shoulder centre. All patients received an uncemented humeral short-stem component (Ascend Monolithic, Wright Medical) in combination with a cemented keeled glenoid component (Aequalis Glenoid, Wright Medical). This implant corresponds to the first generation of the Aequalis short-stem shoulder system; the titanium stem has no proximal porous coating.

Inclusion criteria were: (1) diagnosis of primary glenohumeral osteoarthritis, (2) clinically intact rotator cuff without evidence of higher-grade or full-thickness cuff lesions, (3) implantation of the same short-stem shoulder prosthesis, (4) availability of written informed consent, and (5) a minimum follow-up of nine years with predefined clinical and radiographic evaluations. Exclusion criteria were inflammatory arthritis, post-traumatic or secondary osteoarthritis, relevant previous surgery of the affected shoulder, and higher-grade or full-thickness rotator cuff lesions. Preoperative glenoid morphology was not used as an exclusion criterion; however, a detailed preoperative Walch classification was not consistently available and therefore was not analysed. Follow-up examinations were conducted by an independent investigator not involved in patient care. The local ethics committee provided ethical approval (approval code S-117/2023). The study was conducted in accordance with the Declaration of Helsinki.

Seven patients had died before the final follow-up, and five were lost to follow-up. Consequently, 23 of 28 shoulders (82.1%) were available for long-term clinical and radiological evaluation. The study cohort comprised 12 women (54.5%), one of whom underwent bilateral shoulder arthroplasty, and ten men (45.5%). The mean age at surgery was 68.0 years (range, 58.4–79.0). The right side was affected in 14 shoulders (60.9%) and the dominant arm in 15 cases (65.2%).

### Implant design and surgical technique

The Ascend Monolithic is a titanium short-stem implant without proximal porous coating, designed for metaphyseal fixation. All procedures were performed through a standard deltopectoral approach. Implant size was determined intraoperatively based on metaphyseal press-fit and rotational stability. The glenoid was prepared by reaming and implanted with a keeled, cemented polyethylene component.

All patients followed a standardised postoperative rehabilitation protocol. The arm was immobilised in an abduction pillow day and night until the end of the fourth postoperative week, with removal only for hygiene, physiotherapy, resting on a pillow, and controlled self-exercises. During the first two weeks, therapy focused on cautious passive pain-free mobilisation in the scapular plane with maximum flexion of 90°, abduction of 60°, internal rotation of 45° and external rotation of 10°, together with active hand and elbow exercises, light pendulum exercises, scapular mobilisation and activities-of-daily-living training. During weeks three to six, passive mobilisation was continued with gradual transition to assisted mobilisation; active mobilisation was introduced from week four, and inpatient or outpatient rehabilitation was initiated. During weeks seven to 12, therapy focused on active mobilisation, coordination training, and progressive rotator cuff and scapular strengthening. From week 12 onward, strengthening and activities-of-daily-living training were intensified, and return to sports or other intensive shoulder loading was allowed gradually from week 21 onward.

### Clinical examination

Clinical assessments were performed at five time points: preoperatively (T–1), immediately postoperatively (T0), at early follow-up (T1: 2.1 ± 0.8 years), mid-term follow-up (T2: 4.6 ± 1.0 years), and long-term follow-up (T3: 10.3 ± 1.1 years). Outcome measures included the Constant Score (CS), the age- and sex-adjusted Constant Score (AGACS), pain (assessed as the pain subscore of the CS ranging from 0–15), and the Subjective Shoulder Value (SSV) [12]. Active range of motion (ROM), including forward elevation, abduction, and external rotation, was measured using a goniometer. At final follow-up (T3), isolated glenohumeral abduction and the Disabilities of the Arm, Shoulder, and Hand (DASH) score were additionally assessed [13].

### Radiographic analysis

**Fig. 1:**
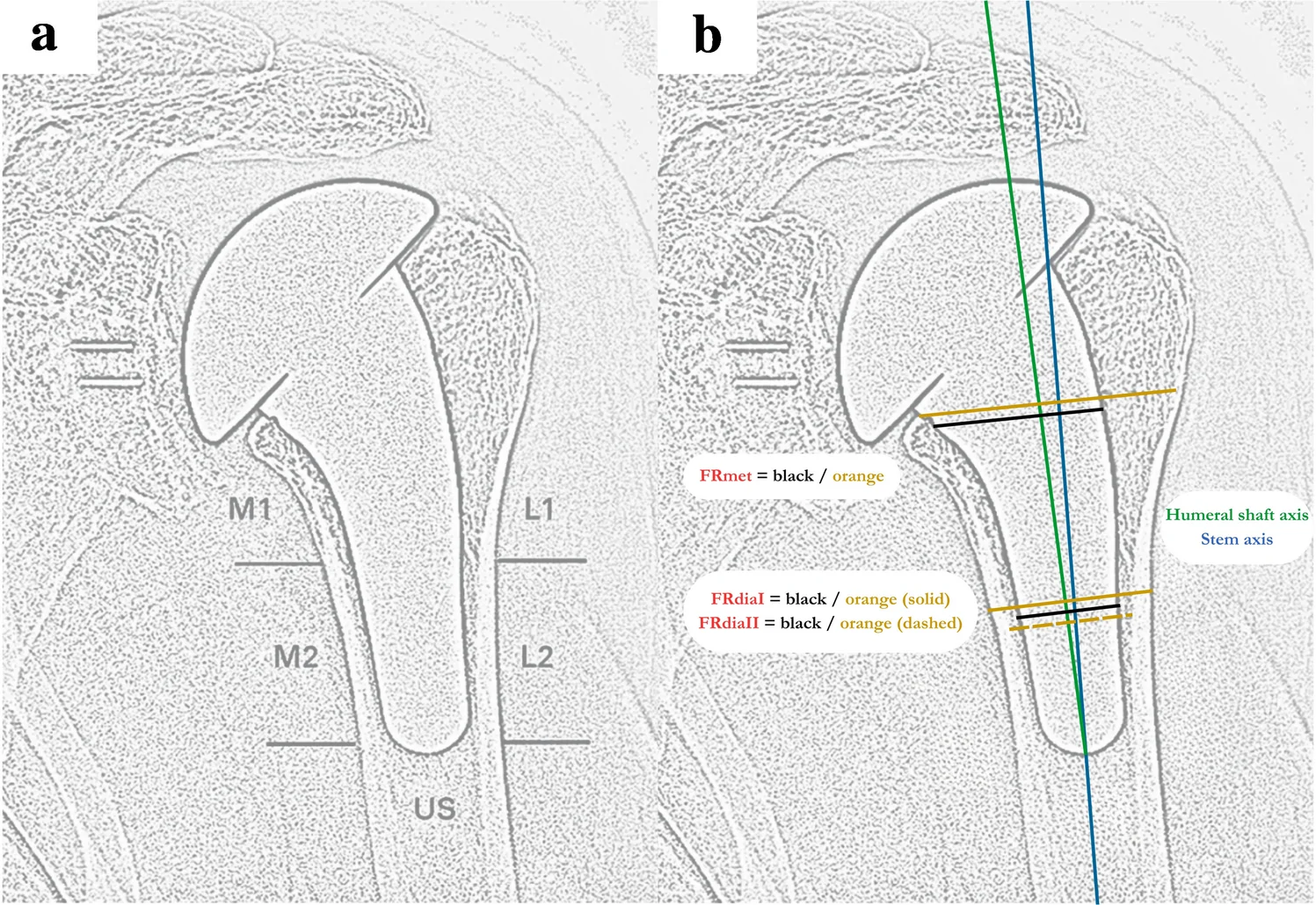
**A**, Five zones around the humeral stem; **B**, Measurement procedures for stem alignment and fill ratios. The inclination angle in anatomic shoulder arthroplasty was defined as the angle between the humeral shaft axis (green line) and the stem axis (blue line). Filling ratios were assessed at two levels: metaphyseal (FRmet) and diaphyseal (FRdiaI and FRdiaII). FRmet was determined using a line perpendicular to the humeral shaft axis drawn at the medial-inferior border of the humeral head. FRdiaI and FRdiaII were measured with perpendicular lines to the humeral shaft axis at the distal third of the stem, referencing the outer and inner cortical boundaries, respectively. At each level, the filling ratio was calculated as the quotient of the stem diameter (black line) and the corresponding humeral diameter (orange line)

At each follow-up, standardized radiographs were obtained, including a true anteroposterior (AP) view and an axillary view of the operated shoulder; only minor rotational deviations were accepted. True AP radiographs at the four postoperative time points (T0–T3) were analyzed for bone adaptation in five zones around the humeral stem, as defined by Nagels and adapted for short-stem designs by Schnetzke et al. (Fig. 1a) [9, 14]. Bone adaptation in the five zones was assessed for three features: condensation lines (CL), cortical narrowing/osteopenia (CNO), and spot welds (SW). All radiographic variables were evaluated by two blinded observers, and discrepancies were resolved by consensus. A formal interobserver reliability analysis was not performed. Features were categorized as: none (0–1 feature), mild (2–3 features), moderate (4–6 features), or severe (≥ 7 features). If both observers identified the pattern as “aggressive,” bone adaptation was classified as severe, independent of the number of features present. For overall classification, adaptations were grouped as low (none/mild) or high (moderate/severe). In addition, a humeral change score was calculated according to Denard et al., summing all radiographic changes across zones [8]. Medial calcar osteolysis was graded separately as none, partial, or complete [11]. At T0 and T3, additional parameters were analyzed in detail: stem inclination (in degrees), filling ratio at metaphyseal and diaphyseal levels, and cortical contact of the stem. Malalignment was defined as a deviation of ≥ 5° from the humeral shaft axis. Because of anatomical variability in diaphyseal cortical thickness, the diaphyseal filling ratio was calculated separately with reference to the outer (FRdiaI) and inner (FRdiaII) cortical boundaries. Measurement procedures for stem alignment and fill ratios are illustrated in Fig. 1b.

Glenoid radiolucencies were scored using the Lazarus and Matsen 0-to-5 grading system for keeled glenoid components, which builds on the radiographic criteria for glenoid loosening described by Franklin et al., and were classified as low (grades 0–1) or high (grades 2–5) [15, 16].

Rotator cuff integrity was indirectly evaluated using the acromiohumeral distance (AHD), measured from the inferior acromion to the superior humeral head component. An AHD ≤ 7 mm was defined as abnormal, indicating humeral head escape, tuberosity malunion, or rotator cuff failure [17].

### Statistics

Statistical analyses were performed using GraphPad Prism (version 10 for macOS). Means, standard deviations, and ranges were calculated. Changes between preoperative and postoperative continuous variables were analyzed with the Wilcoxon signed-rank test. Subgroup analyses of humeral and glenoid bone adaptations were performed using the Mann–Whitney U test. A p-value of ≤ 0.05 (two-sided) was considered statistically significant.

## Results

### Clinical results

Clinical outcomes are summarized in Table 1. The mean Constant Score improved from 29.5 ± 5.6 preoperatively to 65.0 ± 12.2 at early follow-up (T1) and 72.4 ± 7.4 at final follow-up (T3). Significant improvements in CS, AGACS, SSV, pain, and active ROM were observed between the preoperative assessment (T–1) and early follow-up (T1). From T1 to final follow-up (T3), further significant improvements were seen in CS and SSV. Between mid-term (T2) and long-term follow-up (T3), all clinical parameters remained stable without significant changes. According to the Constant Score at final follow-up, an excellent outcome (CS ≥ 70) was achieved in 16 shoulders (69.6%), a very good outcome (CS 60–69) in five shoulders (21.7%), a good outcome (CS 40–59) in one shoulder (4.3%), and a poor outcome (CS < 40) in one shoulder (4.3%).

**Table 1:** Clinical outcome summary

	T_−1_	T_0_	T_1_	T_2_	T_3_	T1vsT-1*	T3vsT1*	T3vsT2*
Mean CS (SD)	29.5 ± 5.6	43.3 ± 8.9	65.0 ± 12.2	70.0 ± 10.3	72.4 ± 7.4	** < 0.0001**	**0.0057**	0.2411
Mean AGACS (SD)	38.9 ± 7.8	57.6 ± 12.4	87.8 ± 20.0	81.5 ± 16.2	88.7 ± 16.6	** < 0.0001**	0.9492	0.3750
Mean SSV (SD)	44.8 ± 13.4	69.0 ± 14.6	87.5 ± 10.1	90.5 ± 8.7	92.7 ± 6.2	** < 0.0001**	**0.0426**	0.2866
Mean pain score (SD)	5.4 ± 2.8	13.5 ± 1.4	14.2 ± 1.0	14.0 ± 1.7	14.3 ± 1.1	** < 0.0001**	0.6892	0.1753
Mean FE, ° (SD)	104.8 ± 21.5	103.6 ± 27.2	158.3 ± 18.3	163.0 ± 12.0	160.4 ± 18.7	** < 0.0001**	0.5518	0.5706
Mean abduction, ° (SD)	90.9 ± 25.9	90.5 ± 27.3	153.0 ± 26.7	156.8 ± 24.2	156.5 ± 23.3	** < 0.0001**	0.4990	0.7172
Mean ER, ° (SD)	3.9 ± 9.4	27.3 ± 21.4	38.7 ± 22.0	42.3 ± 23.1	41.7 ± 22.9	** < 0.0001**	0.2625	0.3357

*CS* Constant Score, *AGACS* age- and sex-adjusted Constant Score, *SSV* Subjective Shoulder Value, *FE* forward elevation, *ER* external rotation; *, Wilcoxon signed-rank test; Bold, statistical significance

**Fig. 2:**
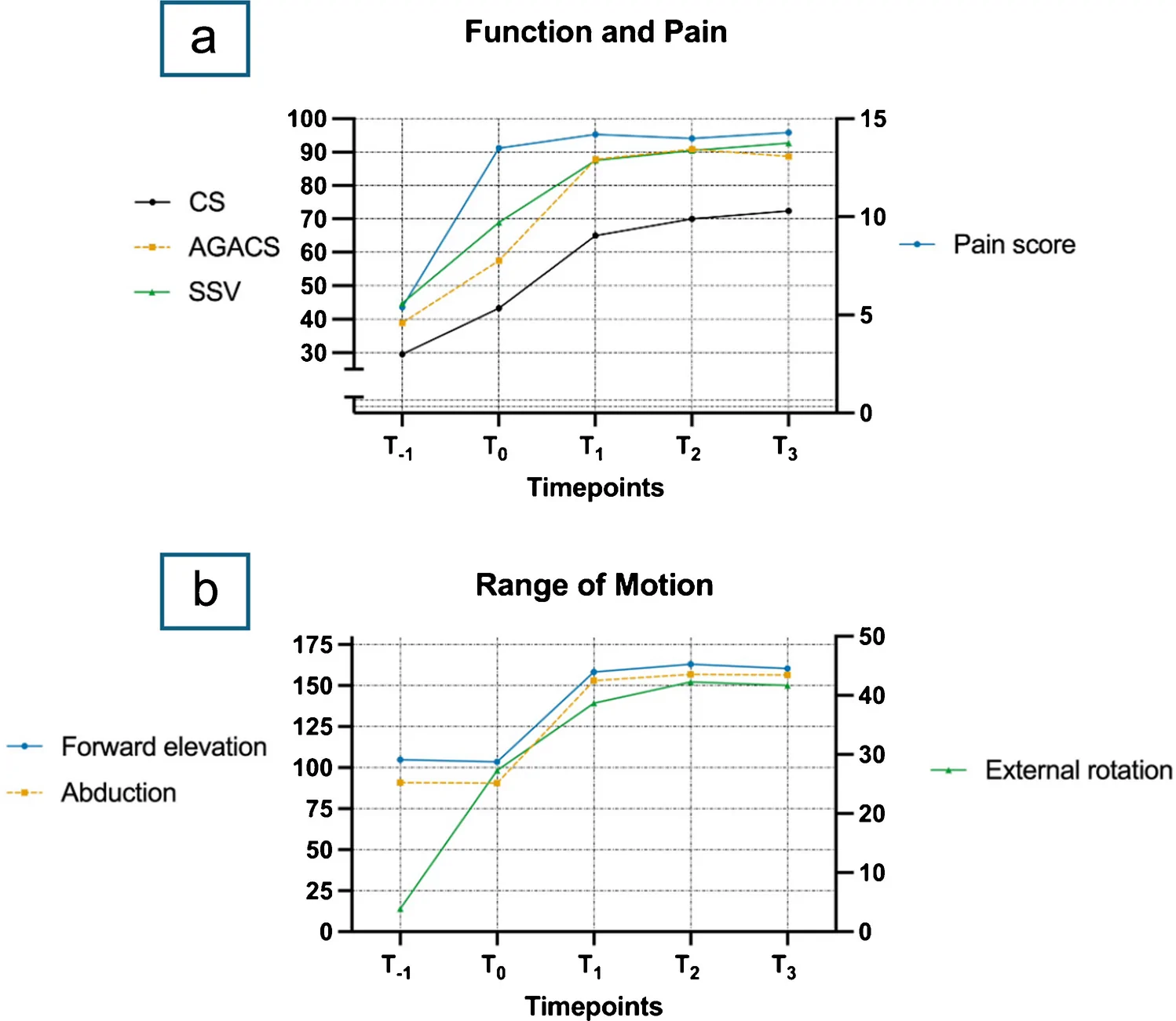
**A**, Functional outcome scores and pain development over time; **B**, Mean range of motion over time. CS, Constant score; AGACS, age- and sex-adjusted Constant Score; SSV, Subjective Shoulder Value

At final follow-up, mean glenohumeral abduction amounted to 86.5 ± 5.7°, and the mean DASH score was 16.0 ± 16.4. Functional outcome scores and pain development are illustrated in Fig. 2a. The progression of mean ROM over time is presented in Fig. 2b.

### Radiographic results

At T0, stem inclination was anatomical in 18 patients and valgus in five patients, with a mean alpha angle of –1.5° (valgus) ± 3.5°. At T3, stem inclination remained anatomical in 17 patients, valgus in the same five patients as at T0, and varus in one patient, with a mean alpha angle of –1.3° (valgus) ± 3.6°. At final follow-up, the mean filling ratios were 0.61 ± 0.08 for FRmet, 0.59 ± 0.05 for FRdiaI, and 0.75 ± 0.08 for FRdiaII. The mean acromiohumeral distance (AHD) decreased from 12.5 ± 3.2 mm at T0 to 10.7 ± 3.1 mm at T1, 9.8 ± 2.8 mm at T2, and 9.1 ± 3.1 mm at T3. In seven patients (30.4%), the AHD measured ≤ 7 mm at final follow-up, indicating secondary rotator cuff insufficiency. The humeral change score increased from 0 at T0 to 2.0 ± 1.5 at T1, 2.7 ± 1.6 at T2, and 3.2 ± 1.9 at T3. Glenoid radiolucency progressed from 0 at T0 to 0.8 ± 1.1 at T1, 1.0 ± 1.2 at T2, and 1.6 ± 1.8 at T3. At final follow-up, humeral bone adaptation was classified as low in 12 shoulders (52.2%) and high in 11 shoulders (47.8%). Distribution of humeral remodeling changes is shown in Table 2.

**Table 2:** Distribution of humeral remodeling changes

	L1	M1	L2	M2	US
T_1_, n (%)					
None	23 (100.0)	12 (52.2)	8 (34.8)	12 (52.2)	21 (91.3)
CL	0 (0,0)	0 (0.0)	1 (4.3)	1 (4.3)	2 (8.7)
CNO	0 (0.0)	12 (52.2)	6 (26.1)	3 (13.0)	0 (0.0)
SW	0 (0.0)	0 (0.0)	12 (52.2)	8 (34.8)	0 (0.0)
T_2_, n (%)					
None	22 (95.7)	7 (30.4)	6 (26.1)	7 (30.4)	17 (73.9)
CL	1 (4.3)	0 (0.0)	1 (4.3)	1 (4.3)	4 (17.4)
CNO	0 (0.0)	14 (60.9)	7 (30.4)	3 (13.0)	0 (0.0)
SW	0 (0.0)	2 (8.7)	14 (60.9)	14 (60.9)	2 (8.7)
T_3_, n (%)					
None	21 (91.3)	8 (34.8)	5 (21.7)	6 (26.1)	13 (56.5)
CL	1 (4.3)	1 (4.3)	1 (4.3)	1 (4.3)	7 (30.4)
CNO	1 (4.3)	13 (56.5)	10 (43.5)	6 (26.1)	0 (0.0)
SW	0 (0.0)	2 (8.7)	14 (60.9)	14 (60.9)	3 (13.0)

*CL* condensation lines, *CNO* cortical narrowing and osteopenia, *SW* spot welds

**Fig. 3:**
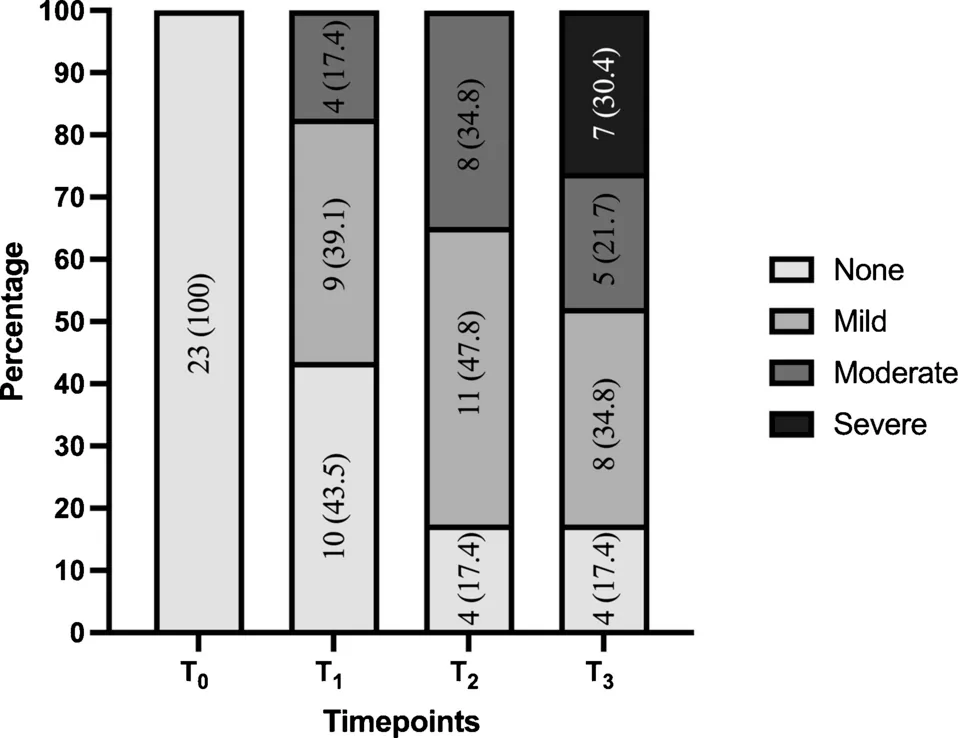
Radiographic changes and their corresponding proportions. CL, condensation lines; CNO, cortical narrowing and osteopenia; SW, spot welds; Numbers indicate absolute cases; percentages are given in brackets

Figure 3 reports the proportions of mild, moderate, and severe humeral remodeling.

At final follow-up, the low and high humeral bone adaptation subgroups comprised 12 and 11 shoulders, respectively, and the low and high glenoid radiolucency subgroups comprised 12 and 11 shoulders, respectively. Comparison between patients with low versus high humeral bone adaptations at final follow-up (Table 3) revealed a significant difference in AHD (13.3 ± 1.7 mm versus 11.6 ± 4.2 mm; p = 0.0145), whereas functional outcomes were unaffected by the degree of remodeling. Filling ratios were comparable between the low and high humeral bone adaptation groups (FRmet: 0.62 ± 0.08 versus 0.59 ± 0.07, p = 0.2510; FRdiaI: 0.59 ± 0.05 versus 0.60 ± 0.05, p = 0.3532; FRdiaII: 0.75 ± 0.08 versus 0.76 ± 0.08, p = 0.8680). Comparisons of patients with low versus high glenoid radiolucency (Table 3) showed that higher radiolucency was associated with reduced forward elevation and abduction at final follow-up.

**Table 3:** Clinical and radiographic outcomes according to humeral bone adaptation and glenoid radiolucency at final follow-up

	Low humeral bone adaption (*n* = 12)	High humeral bone adaption (*n* = 11)	*p*-value*
Mean CS (SD)	72.7 ± 7.8	72.2 ± 7.4	0.8918
Mean AGACS (SD)	91.5 ± 16.0	85.5 ± 17.3	0.3834
Mean SSV (SD)	92.8 ± 7.0	92.7 ± 5.6	0.8916
Mean pain score (SD)	14.2 ± 1.1	14.4 ± 1.2	0.6113
Mean FE, ° (SD)	165.0 ± 6.7	155.5 ± 25.8	0.5492
Mean abduction, ° (SD)	162.5 ± 14.2	150.0 ± 29.7	0.4041
Mean ER, ° (SD)	45.8 ± 23.1	27.3 ± 22.8	0.2223
FRmet	0.62 ± 0.08	0.59 ± 0.07	0.2510
FRdia I	0.59 ± 0.05	0.60 ± 0.05	0.3532
FRdia II	0.75 ± 0.08	0.76 ± 0.08	0.8680
AHD	13.3 ± 1.7	11.6 ± 4.2	**0.0145**
	**Low glenoid radiolucencies (n = 12)**	**High glenoid radiolucencies (n = 11)**	
Mean CS (SD)	73.0 ± 7.9	71.8 ± 7.2	0.5340
Mean AGACS (SD)	88.7 ± 17.3	88.6 ± 16.5	0.9857
Mean SSV (SD)	91.7 ± 6.5	93.9 ± 6.0	0.4116
Mean pain score (SD)	14.2 ± 1.1	14.4 ± 1.2	0.6113
Mean FE, ° (SD)	168.3 ± 5.8	151.8 ± 24.0	0.0198*
Mean abduction, ° (SD)	166.7 ± 9.8	145.5 ± 28.8	0.0283*
Mean ER, ° (SD)	42.5 ± 22.2	40.9 ± 24.7	0.7026

*CS* Constant Score, *AGACS* age- and sex-adjusted Constant Score, *SSV* Subjective Shoulder Value, *FE* forward elevation,, *ER* external rotation, *AHD* acromiohumeral distance; *, Mann–Whitney U test

### Complications

**Fig. 4:**
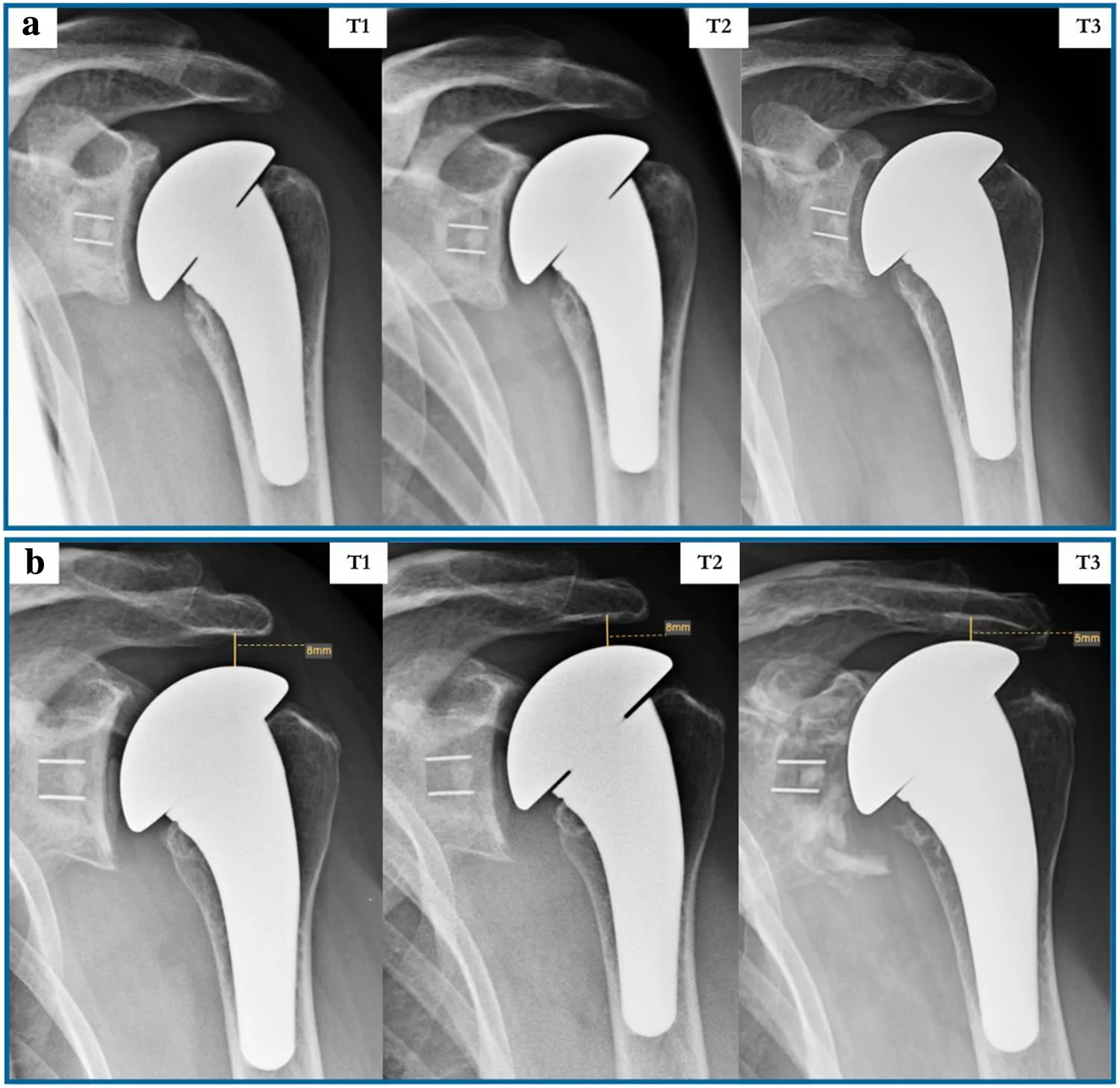
Serial radiographs showing a normal course and progression from early radiolucency to gross glenoid loosening. **a, upper row:** Over time, only remodeling of a partial medial calcar osteolysis is observed (from T2 to T3). The humeral stem shows no cortical contact at any time, and no proximal migration is evident. This represents a normal postoperative course. **b, lower row:** At T1, a radiolucency at the inferior flange of the glenoid component is visible (Lazarus grade 1), with cortical contact of the humeral stem and an acromiohumeral distance of 8 mm. At T2, radiolucencies are present around the glenoid keel but remain < 2 mm (Lazarus grade 3), with unchanged acromiohumeral distance. At T3, gross glenoid loosening is observed (Lazarus grade 5), accompanied by cortical bone adaptations in M1, L2, M2, and US, and at least partial osteolysis of the medial calcar. The acromiohumeral distance is reduced to 5 mm, indicating secondary rotator cuff insufficiency in the setting of aseptic glenoid loosening

One patient (4.3%) required revision surgery 13.2 years after the index procedure due to aseptic loosening of the glenoid component (Fig. 4). Radiographic follow-up demonstrated progressive radiolucency, graded as 1 at the two-year visit and 3 at five years, which advanced to grade 5 at final follow-up. The shoulder was converted to a reverse total shoulder arthroplasty.

## Discussion

### Clinical outcomes

The present study demonstrates that aTSA with a cementless short-stem humeral component provides sustained clinical improvement over a decade of follow-up. The mean CS improved from 29.5 ± 5.6 preoperatively to 72.4 ± 7.4 at final follow-up, and 70% of shoulders achieved excellent outcomes. These findings are consistent with early- to mid-term reports of short-stem implants, which demonstrated substantial gains in pain relief, function, and ROM [9, 18–21]. While many previous studies reported only short- or mid-term outcomes, our data extend these findings into the long term, showing that improvements remained stable beyond ten years in this cohort. Because the present study had no comparative control group, these results should be interpreted as supportive evidence for durable clinical benefit rather than evidence of superiority or equivalence compared with other stem designs [22, 23].

### Radiographic bone adaptations

Radiographic analysis revealed frequent metaphyseal and calcar adaptations, including CNO, CL, and SW, with prevalence similar to earlier reports on the same implant family [20, 22, 24]. In our series, bone adaptations did not correlate with inferior clinical results, which is reassuring. However, given the limited cohort size and the absence of a comparator, this should not be interpreted as definitive evidence that these radiographic changes are biologically or mechanically benign in the long term. Comparative studies have suggested that shorter stems reduce stress shielding compared to traditional-length stems [5, 25]. In the present cohort, no humeral component loosening was detected despite the high prevalence of radiographic changes.

### Rotator cuff integrity and acromiohumeral distance

At final follow-up, reduced AHD (≤ 7 mm) was observed in seven patients (30.4%), suggesting secondary rotator cuff insufficiency. Because rotator cuff integrity was assessed indirectly by AHD rather than systematically by ultrasound or magnetic resonance imaging, the structural accuracy of this assessment is limited. Comparable long-term series report high rates of radiographic superior migration after aTSA [26]. Lafosse et al. noted superior migration in 44% of patients at ≥ ten years, yet these findings were not associated with inferior clinical outcomes and ten year implant survival remained 89% [26]. The clinical relevance of cuff integrity after long-term aTSA is highlighted in the literature, as progressive superior migration has been correlated with worse functional outcomes and increased revision risk [27, 28].

### Glenoid component survival

The glenoid component remains a potential weak link of aTSA. In our cohort, one patient required revision for aseptic loosening after 13.2 years. This revision was preceded by progressive radiographic changes, evolving from grade 1 at two years to grade 5 at the time of revision. Glenoid loosening is the most common late complication after aTSA, with studies reporting revision rates between 5 and 15% at ten to 15 years [26, 29]. The revision rate in the present cohort was low, but this finding should be interpreted cautiously because of the small sample size and the single revision event. Radiographic radiolucency and loosening remain relevant concerns, as highlighted by large registry and cohort studies [10, 27]. The present data therefore suggest an association between higher glenoid radiolucency and inferior range of motion, but they do not establish a causal relationship.

### Complications and revisions

Overall, complications were infrequent in this cohort. One revision was performed for glenoid loosening and secondary rotator cuff failure. No humeral revisions occurred, which is notable given concerns about humeral loosening in some short-stem designs [21, 30]. Because only one revision event occurred, a formal Kaplan–Meier survivorship analysis would have been statistically unstable and was therefore not performed.

### Biomechanical considerations

Finite element analyses provide a mechanistic rationale for the observed clinical stability of short-stem humeral components. Stress distributions with short stems more closely approximate the intact humerus compared to long stems, particularly in proximal cortical bone [5, 25]. However, excessive filling ratios or malalignment may predispose to bone resorption and high-grade remodeling [20, 22]. In our cohort, more pronounced humeral bone adaptations had no significant impact on clinical outcomes, and higher grades of remodeling were not associated with increased filling ratios, in contrast to previous reports [24]. Potential explanations include the absence of proximal porous coating in this implant design, subtle patient-specific differences in stem fit or bone quality, and limited statistical power.

### Limitations

Strengths of this study include its long follow-up, consistent surgical technique by a single surgeon, and systematic clinical and radiographic evaluation at multiple intervals. Nonetheless, several limitations must be acknowledged. First, the study represents a retrospective single-centre series with a limited sample size, which restricts the external validity of the findings. Second, although follow-up of over 80% of surviving patients was achieved, attrition due to death, comorbidity, or loss to follow-up may have introduced survivorship bias. Third, preoperative glenoid morphology according to the Walch classification was not consistently available and therefore could not be analysed, although glenoid morphology may influence loosening risk and long-term outcome after aTSA. Fourth, rotator cuff integrity was assessed indirectly using AHD without systematic advanced imaging, which limits the accuracy of structural cuff evaluation. Fifth, formal interobserver reliability statistics such as Cohen’s kappa or intraclass correlation coefficients were not calculated. Sixth, because only one revision event occurred, no robust Kaplan–Meier survivorship analysis was performed. Seventh, the use of plain radiographs limits the precision of evaluating bone adaptations compared to CT-based methods [31]. Finally, no adjustment for multiple testing was performed; therefore, the reported p-values should be interpreted in a descriptive and exploratory manner.

## Conclusion

This retrospective case series indicates that anatomic total shoulder arthroplasty with a cementless short-stem humeral component can provide sustained clinical benefit, reliable humeral fixation, and acceptable complication rates at a mean ten-year follow-up. While radiographic bone adaptations were common, they were not associated with inferior function or humeral implant failure in this cohort. Glenoid radiolucency and secondary rotator cuff insufficiency appeared to be relevant long-term concerns, although causal conclusions are limited by the small cohort size and observational design. These findings support continued clinical and radiographic surveillance after short-stem aTSA and further comparative long-term studies.

## Data Availability

No datasets were generated or analysed during the current study.
